# Surveillance of Individuals with a Family History of Pancreatic Cancer and Inherited Cancer Syndromes: A Strategy for Detecting Early Pancreatic Cancers

**DOI:** 10.3390/diagnostics9040169

**Published:** 2019-10-31

**Authors:** Hiroyuki Matsubayashi, Yoshimi Kiyozumi, Hirotoshi Ishiwatari, Katsuhiko Uesaka, Masataka Kikuyama, Hiroyuki Ono

**Affiliations:** 1 Division of Endoscopy, Shizuoka Cancer Center, Shizuoka 411-8777, Japan; h.ishiwatari@scchr.jp (H.I.); h.ono@scchr.jp (H.O.); 2 Division of Genetic Medicine Promotion, Shizuoka Cancer Center, Shizuoka 411-8777, Japan; y.kiyozumi@scchr.jp; 3 Division of Hepato-Biliary-Pancreatic Surgery, Shizuoka Cancer Center, Shizuoka 411-8777, Japan; k.uesaka@scchr.jp; 4 Department of Gastroenterology, Tokyo Metropolitan Cancer and Infectious Diseases Center Komagome Hospital, Tokyo 113-0021, Japan; kikuyama110@yahoo.ne.jp

**Keywords:** familial pancreatic cancer, genetic, high risk, surveillance, treatment

## Abstract

A family history of pancreatic cancer (PC) is a risk factor of PC, and risk levels increase as affected families grow in number and/or develop PC at younger ages. Familial pancreatic cancer (FPC) is defined as a client having at least two PC cases in a first degree relatives. In the narrow sense, FPC does not include some inherited cancer syndromes that are known to increase the risks of PC, such as Peutz–Jeghers syndrome (PJS), hereditary pancreatitis (HP), hereditary breast ovarian cancer syndrome (HBOC), and so on. FPC accounts for 5%–10% of total PC diagnoses and is marked by several features in genetic, epidemiological, and clinicopathological findings that are similar to or distinct from conventional PC. Recent advances in genetic medicine have led to an increased ability to identify germline variants of cancer-associated genes. To date, high-risk individuals (HRIs) in many developed countries, including FPC kindreds and inherited cancer syndromes, are screened clinically to detect and treat early-stage PC. This article highlights the concept of FPC and the most recent data on its detection.

## 1. Introduction

Pancreatic cancer (PC) is one of the most aggressive forms of cancer in humans. The overall prognosis is quite poor, and the five-year survival rate for cases of PC is 13%. However, resection while the carcinoma is still limited to a minimal invasion (≤10 mm of tumor size) improves the five-year survival to 80% [1]. Hence, early detection and suitably timed resection are the ideal strategy to tackling PC. Recently, the possibility of detecting early PC has increased, following incidental findings uncovered during the clinical management of other diseases [2]. Early PC detection has also been achieved through screenings of high-risk individuals (HRIs), such as family members of PC patients and those with inherited cancer syndromes [3].

According to some previous case-controlled and cohort studies, it is clear that first degree relatives (FDRs) of PC patients face an increased risk of developing PC themselves (2.1 [4] –5.3 [5] odds ratio (OR) and 1.5 [6] –1.7 [7] relative risk (RR)) [8]. The incidence of PC rises with the number of family members with PC (standardized incidence ratio (SIR): 4.5 in families with one FDR of PC, 6.4 in two FDRs, and 32 in ≥3 FDRs) [9]. In a general sense, having two or more PC patients as FDRs is a state defined as familial pancreatic cancer (FPC) [10], which accounts for 5–10% of all PC cases. In a more narrow sense, known genetic syndromes are excluded from this condition [9] (see Supplementary Table S1), these include Peutz–Jeghers syndrome (PJS) [11], hereditary pancreatitis (HP) [12], familial atypical multiple mole melanoma (FAMMM) [13,14], hereditary breast-ovarian cancer (HBOC) [15,16], Lynch syndrome (LS) [17,18], and familial adenomatous polyposis (FAP) [19].

Since the 1990s, many countries and institutions have established FPC registries, and clinical observational studies have been concluded on the early detection and cure of PCs that develop among HRIs.

## 2. Characteristics of FPC

### 2.1. Epidemiology

FPC is remarkable for epidemiological characteristics relative to ordinary PC. As similar to sporadic PC (SPC) cases, smoking [8,20] and diabetes [8] are both risk factors for FPC, and an earlier onset of the disease is common among smokers in FPC (the typical onset for smokers is 63.7 years, while it is 66.6 years for non-smokers, *p* = 0.05) [20]. Ethnic deviations have been a concern for Ashkenazi Jews, as they represent a genetically distinct population [21] characterized by a higher than average prevalence of germline variants in *BRCA1*, *BRCA2*, *MSH2*, and *MSH6* [22,23]. Upon closer review of New York City death certificates, it becomes clear that there is a higher mortality rate due to PC among Jewish groups than non-Jews (RR: 1.43, to compared with non-Jewish patients). Despite the above, a recent study from Harvard University has identified Ashkenazi Jews’ hazard ratio (HR) by family history of PC as 2.79 [24], which is not remarkable when compared with worldwide data [25]. Another recent study that analyzed the Utah Population Database found 4095 cases of PC and 40,933 controls. The database revealed a higher risk of PC among the FDRs of female PC patients (RR: 1.96) and in the FDRs of younger PC patients (<65 years, RR: 2.12) [26]. The lifetime risk of PC increases with the decreasing age of PC onset in family members [27,28], while the SIR (9.31) in members of FPC kindreds with young onset (<50 years) PC is higher than those without (SIR: 6.34) (*p* < 0.001). Two European FPC registries [29,30,31], the European Registry of Hereditary Pancreatitis and Familial Pancreas Cancer (EUROPAC) and the German National Case Collection for Familial Pancreatic Carcinoma (FaPaCa), analyzed 106 FPCs over three generations and observed a trend of younger onset and worse prognoses among the youngest generation [32].

### 2.2. Pathology

The histology of the pancreas in FPC kindreds often demonstrates multiple precancerous lesions [33], including pancreatic intraepithelial neoplasm (PanIN) and intraductal papillary mucinous neoplasm (IPMN) [34,35]. These precancerous lesions were more often recognized in PFCs’ pancreases than in SPC patients (2.8-fold, *p* < 0.05); this is a remarkable trend for incipient IPMNs (11.8-fold) [36]. The pancreases of FPC kindred are sometimes associated with parenchymal atrophy and early chronic pancreatitis changes, which can be observed via endoscopic ultrasonography (EUS) [35].

Despite the differences in these precursor lesions [33,35], a blind analysis of histological observations by expert pathologists found no significant differences between the cancerous tissues of 519 FPCs and 561 SPCs in terms of their location, lymph node metastasis, neural invasion, pathological stage, tumor size, or vessel permeation [37].

### 2.3. Molecular Biology

The genome-wide allelic status [38,39], genetic (somatic mutation of K-*ras*, *TP53*, and *DPC4*), and epigenetic (promoter methylation of *SPARC*, *NPTX2*, *CDKN2A*, etc.) alterations frequently observed in PC [40] have been compared between SPCs and FPCs, but no obvious difference has yet been recognized.

### 2.4. Germline Variants

Unlike other familial tumors, germline pathogenic variants have been proven in fewer than 20% of FPC cases [3]. Recently, variants of genes functioning in the homologous recombination (HR) pathway have been considered not only in terms of surveillance, but also as they relate to treatments [41]: *ATM* (variant rate: 2–4%) [42,43], *BRCA1* (0–7%) [44,45], *BRCA2* (4–17%) [15,43,46], *CHEK2* (1–6%) [47], *PALB2* (1–4%) [43,48,49], and *RAD51* (4%).

Carriers of *BRCA1/2* variants have a modest risk for PC (relative risks: 2–8%; lifetime risks: 2–17%), but other specific variants have greater increased risks. For instance, *BRCA2* 6174delT, a Jewish founder variant, was detected in 13% of Jewish PC cases (OR: 12.8 [50]). The *BRCA2* K3326X variant was detected in 5.6% of 144 American FPC cases, a significantly more common rate than among SPCs [51]. A murine experimental model demonstrated that a germline *BRCA2* variant [52] promoted carcinogenesis via the K-ras mutation [53], which confirms the function of the *BRCA2* mutation in FPC. Apart from these genes associated with the HR pathway, variants of those genes responsible for several inherited cancer syndromes (see Supplementary Table S1) are also causative for FPCs, in a general sense.

Recently, precision medicine, a new cancer treatment strategy, has been applied to the treatment of advanced cancers. One result has been that unexpected variants of cancer-associated genes are now detected as increasing rates [54]. Commercially available tests also include a panel of genes known to be causative for FPCs. Above all, variants of HR-associated genes [42,43,44,45,46,47,48,55] and mismatch repair genes [56] are often recognized and related to the drug choice (poly ADP-ribose polymerase (PARP) inhibitor [41] and immune checkpoint inhibitor [57], respectively).

## 3. Clinical Managements for the Individuals with Inherited Risk of PC

### 3.1. FPC Registries

The notion of a FPC registry emerged with the establishment of the National Familial Pancreas Tumor Registry (NFPTR) at Johns Hopkins University (Baltimore, UUS), in 1994 [58]. This was soon followed by the EUROPAC, in 1997 [29], at Liverpool University, in the UK, and the FaPaCa [30] at Phillips University, in Marburg, Germany, in National FPC registries have also been established in Italy (2007) [59], Spain (2009) [60], Australia (2011), and Japan (Japanese Familial Pancreatic Cancer Registry, JFPCR) (2014) [61].

At present, the JFPCR is planning a prospective cohort study of FPCs and their relatives; its primary goal will be to investigate the etiology of FPC and to clarify the basic and clinical issues Japanese FPCs face. Japanese experts, such as researchers, clinicians, genetic counselors, and statisticians from 21 hospitals nationwide have discussed and proposed a FPC management system. As of January 2019, 98 families and 678 HRIs had been registered with the JFPCR. JFPCR has established the expert consensus for managing the HRIs of PC (2019) and HRIs are recommended to be screened for the pancreas by the combination of image modality and blood test every six months.

### 3.2. Targeted Lesions

Worldwide experts in PC gathered at the International Symposium on Inherited Diseases of the Pancreas [62] and International Cancer of the Pancreas Screening Consortium (CAPS) (2011) [63] to discuss PC screening targets and concluded that prior screening programs for HRIs have aim to detect and treat high-grade precursors (high-grade PanIN [34] and IPMN [64]) or UICC-stage IA PC (T1N0M0) [63]. The five-year survival rate of UICC stage IA cancer is 68.7%. Thus, the ideal targeted lesion is thought to be a high-grade precursor or UICC-stage 0 PC (five-year survival: 85.8%) [1].

### 3.3. High-Risk Individuals (HRIs)

Several consortiums have recommended that those with a 5- [63,65] to 10-fold [62] risk undergo PC screening. Candidates’ risk profiles are determined based on their numbers of affected family members and hereditary syndromes (germline mutated genes) (see Supplementary Table S1). The CAPS consortium proposed nine conditions for screening candidates (see Supplementary Table S2). The risk factors that have been associated with lifestyle and pancreatic diseases should also be taken into consideration as a part of the screening process, such as smoking [8,66], obesity [67,68], physical inactivity [68], diabetes [8,62,69], chronic pancreatitis [62,70,71], IPMN [64], pancreatic cysts [72], pancreatic duct ectasia [72], and so on. For instance, a patient who smokes and has diabetes mellitus with one FDR with PC has a 10-fold risk odd for developing PC, compared to negative controls [8]. Therefore, any counseling provided to HRIs should include information concerning modifiable lifestyle risks, and their improvement should be recommended (i.e., smoking cessation, a healthy diet high in fruits and vegetables, and regular exercise to control weight (body mass index: <25 kg/m^2^) [62].

### 3.4. Timing of Screening Initiation and Intervals

In many institutions, PC screens begin at age 40 [73,74], or 10 years younger than the age of the youngest relative with PC [30,75]. Given that PC develops in cases of PJS at a young age (average onset: 40.8 years) [11], their screenings typically start at 30 [73]. When we consider screenings’ effectiveness, we know that the detection of pancreatic lesions increases after the ages of 50–60 [76], so more than half (51%) of the experts in the CAPS consortium have voted for initial screenings for standard FPC kindreds to begin at age 50 [63].

In addition to the above, many institutions recommend yearly screenings, so long as a patient’s latest pancreas image is normal [63]. However, once an abnormal finding is observed, subsequent screenings are conducted every 3–6 months [31,73,77] or every 3–12 months [63]. Other endorsed screening intervals are: 6–12 months for a non-suspicious cyst; 3 months for a newly detected solid lesion, if surgery is not imminent; and 3 months for an indeterminate main pancreatic duct stricture. FPCs’ natural histories and progressions still require study before judging the appropriate screening intervals based on risk level.

### 3.5. Screening Modalities

Although no full consensus was reached in the CAPS meeting [63], EUS is regarded as the most suitable PC screening modality, based on its ability to detect small pancreatic lesions (<1 cm) [73,78]. EUS is also superior at detecting risk findings frequently seen among HRIs, such as duct ectasia, parenchymal findings of the pancreas [35], and cysts [72]. Drawbacks associated with EUS include the need for a relatively long fasting period and conscious sedation; operator-dependent visualization and interpretation [79]; and a limited observation area, in cases where a patient has a reconstructed upper gastrointestinal tract. In this sense, abdominal ultrasonography is a handy tool that may be substituted for EUS, if the pancreas is well visualized without any blind spots [72]. MRI and magnetic resonance cholangiopancreatography (MRCP) are also useful for visualizing the pancreatic ductal systems. Dilation of the pancreatic duct and cyst formation are risk factors for PC [72] and are frequently recognized among HRIs (cysts in 38.9% and duct ectasia in 2.3%) [76]. EUS and MRI are considered the most accurate imaging tools with high agreement among the consortium experts (agreement, EUS: 83.7% and MRI/MRCP: 73.5%) [63]. In addition to image analyses, serum tumor markers, including carcinoembryonic antigen and cancer antigen 19-9, should be checked at every screening [63,75].

### 3.6. Image findings Among HRIs

When investigating individuals with inherited PC, clinical images of their pancreases show a variety of characteristic findings. On the EUS, HRIs demonstrate several findings often seen in early chronic pancreatitis: cysts, high echoic foci, lobularity, strandings, and a high-echoic margin of the main pancreatic duct (MPD) [80]. However, these EUS findings are not truly specific to FPC kindreds, so any interobserver agreement for these findings is limited to the fair level, even among expert endosonographers (κ <0.4) [81].

Pancreatic investigations using computed tomography (CT), MRI, and EUS conducted in five US academic medical centers demonstrated pancreatic findings in 42% of 216 asymptomatic HRIs; these included cysts (39%, mean: 0.55 cm in size), pancreatic duct dilation (2.3%) and solid mass (1.4%), and an increasing trend by age (14% among those <50 years, 34% in patients 50–59 years, and 53% in 60–69 years; *p* < 0.0001) [76]. A recent study by the EUROPAC also detected 41 (13%) cystic lesions in 321 FPC kindred individuals [82].

A collaborative study by the Leiden FPC Group and FaPaCa demonstrated that pancreatic cystic lesions, or IPMNs, were frequently recognized in FPC kindreds (some of this group carried *BRCA2* and *PALB2* variants) than in *CDKN2A*/*p16* (*p16-Leiden*) variant carriers (42% vs. 16%). Cystic lesions were mostly stable in the FPC cohorts, while the malignant transformation of the cyst was more common (0.8% in the FPC cohort vs. 7% in the *p16-Leiden* cohort) [83]. A similar outcome was observed in a Dutch cohort study (Dutch Research Group on Pancreatic Cancer Surveillance in High-risk Individuals) [84]. They reported that pancreatic cystic lesions (10 mm or greater in size) were more likely to be seen in 88 FPC kindreds without germline variants than in 98 variant carriers (16% vs. 5%, *p* = 0.045). However, pancreatic cysts were significantly more likely to progress in the variant carriers than in the non-carrier group (16% vs. 2%, *p* = 0.05) [84].

IPMNs were sometimes recognized in cases with PJS (*STK11/LKB1* variant) [85] and FAP (*APC* variant) [86]. Variants of cystic fibrosis transmembrane conductance regulator (*CFTR*) [87], serine protease inhibitor Kazal type 1 (*SPINK1*), and cationic trypsinogen (*PRSS1*) [88] develop PC during long-standing chronic pancreatitis, so that PC can be accompanied by severe pancreatic atrophy, pancreatic stones, and duct ectasia. PC with microsatellite instability is reported to be concordant with the medullary growth histology and negative for somatic K-ras mutation [89]. Our previous study showed a unique histology, that of a dendritic structure, of PC in a case with a germline *PTEN* variant [90]. Further data accumulation is needed for solid evidence; however, the image findings among HRIs may vary depending on the associated genetic changes.

### 3.7. Pathological Sampling for the Detection of Early Pancreatic Cancer (Proposal)

EUS-guided fine needle aspiration (EUS-FNA) and endoscopic retrograde cholangiopancreatography (ERCP) are useful in obtaining pathological evidence when abnormal findings are observed in surveillance images [73,74,75]. Hence, clinicians must be cautious about suspicious pancreatic findings, such as pancreatic masses, enhanced nodules, pancreatic duct stenosis/narrowing, and focal pancreatic atrophy [2].

**Figure 1:**
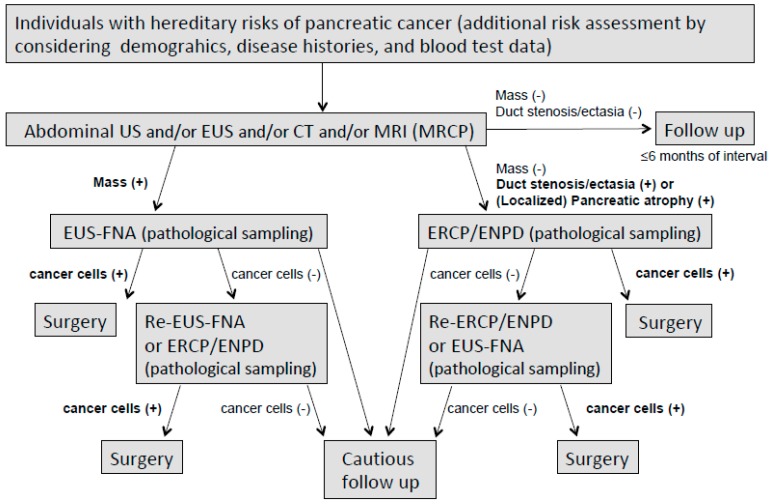
Proposal of surveillance and investigation of individuals with inherited risks of pancreatic cancer.

Our diagnostic strategy is summarized in Figure 1 In cases with a visible pancreatic mass of uncertain malignancy, EUS-FNA is performed even for masses sized ≤1 cm [91,92,93]. In cases of ductal lesions, either stenotic or ectatic, with or without (localized) pancreatic atrophy, the preferred strategy is pancreatic juice extraction for cytology using endoscopic naso-pancreatic ductal drainage (ENPD) [94,95], as small PCs tended to extend intraductally, compared with the larger ones [96]. An ENPD test should be avoided in cases with a high risk of post-ERCP pancreatitis, such as those with rich pancreatic parenchyma, a normal MPD width, pancreatic divisum, the secretion of highly viscous mucus that may stick inside an ENPD, etc. Pre-surgical EUS-FNA from the image-typical cancer lesions at the pancreas’ body and tail are controversial, because of the possibility of cancer seeding [97]. Indications for these examinations should be discussed among experts at each institution prior to their use.

### 3.8. Surgical Indications and Procedures

The extent of cancer-related resections is controversial, depending on the therapeutic concept. In this context, the choices are the removal of all precancerous lesions [75] or the resection of only a targeted area that includes nodular or cystic lesions [73]. In cases of HBOC with the *BRCA* mutation, risk-reducing salpingo-oophorectomy is not only affordable, it also has an acceptable level of complications [98]. However, for the pancreas, prophylactic total pancreatectomy has severe complications, including a considerable level of postsurgical in-hospital mortality (5–23% in Germany) [99,100] and subsequent serious glycemic control failure (mortality: 4–8% per year) [101]. A secondary pancreatectomy for the remnant pancreas can be conducted without increasing morbidity and mortality [102], so resection of the target area, rather than a total pancreatectomy, has been preferable to date. Despite the above, a total pancreatectomy combined with islet autotransplantation has been applied to cases of HP with long-standing pain that has been untreatable by medicine. Even more recently, due to the improvements in post-surgical quality of life, this operation is now indicated for FPC kindred with premalignant lesions [101,103]. Further improvements are expected in the future.

### 3.9. Present Outcomes of Surveillance

Several surveillance results have been reported by Western FPC registries (see Supplementary Table S3) [3,31,73,77,80,104,105,106,107,108,109,110]. Roughly 2–19% of the HRIs screened underwent surgery for suspected lesions. Among these, about one fifth were borderline precursors and carcinoma in situ, or definitive targets of the surveillance, while 30–40% of the resected cases were benign lesions. A small proportion of PC was resected at an early phase (T1N0M0), and some PC cases were detected at the unresectable stage. These outcomes are far from the goal of the surveillance. However, a recent study at Johns Hopkins demonstrated a three-year survival rate of ten PC cases diagnosed during surveillance was 85%, significantly longer than those detected outside the surveillance study (*p* = 0.0009). In addition, all ten cases with high-grade PanIN (PanIN3) or high-grade IPMN were alive after surgery (4.1–14.7 years). These data suggest that recent surveillance systems are improving and prolonging the PC-associated survival rate among HRIs [77].

### 3.10. Application of Blood Circulating Biomarkers for Detecting Early Pancreatic Cancer

Blood circulating biomarkers for detecting early pancreatic cancers have been discovered including circulating tumor DNA [111,112], exome-derived DNA [113], MicroRNA [114] (miR-93, miR-16, miR-548d-3p, etc.), and proteins [115] (SYCN, REG1B, PRSS2, etc.). Liu et al. [112]. reported that 791 cancer-specific cell free DNA fragments with mutations were detected in plasma of 88% of total PC patients and K-ras hotspot mutation detected in 72% of stage I/II PC patients. Allenson et al. [113] reported exome-derived K-ras mutant DNA was detected in 44% of early-stage PC patients and 20% of healthy controls. These promising biomarkers can be applied to the screening of the asymptomatic HRIs of PC.

## 4. Pharmacological Treatments for Familial Pancreatic Cancer

Today, FOLFIRINOX (fluorouracil, folic acid, irinotecan, and oxaliplatin) and gemcitabine-based regimens are standard chemotherapy regimens for unresectable PCs [116]. In cases of advanced PCs with *BRCA* variants, superior overall survival was recognized in the cases treated by platinum-based chemotherapies than those by non-platinum agents (22 months and 9 months, *p* = 0.04) [117]. A similar trend was observed in the progression-free survival after the initiation of oxaliplatin-based chemotherapy in cases with PCs; between PCs with and without somatic mutations of homologous recombination-related genes (20.8 months in mutant group vs. 1.7 months in wild-type group, *p* = 0.049) [118]. In a most recent study, a randomized, double-blind, phase 3 trial (Pancreas Cancer Olaparib Ongoing trial: POLO trial) for germline *BRCA*-variant cases with a metastatic PC, that had not progressed during first-line platinum-based chemotherapy, demonstrated a significantly longer progression-free survival in the oraparib (poly (adenosine diphosphate-ribose) polymerase inhibitor) group (7.4 months) than in the placebo group (3.8 months) (hazard ratio: 0.53, *p* = 0.004), although their overall survivals by an interim analysis were not different between the two groups (median: 18.9 months vs. 18.1 months; hazard ratio: 0.91, *p* = 0.68) [41].

## 5. Conclusions

FPCs have several characteristics that are similar to SPCs, and many others that differentiate them from the group. A family history of PC and certain genetic syndromes should be taken into consideration when screening candidates in hopes of detecting early PC. To date, scientific data regarding FPCs has been gathered via family registries. Genetic information about these HRIs can have an influence on their clinical management, and even on the treatment choice offered to them. The outcomes of HRIs’ screenings have improved in recent decades, but remain unsatisfactory. Further innovation and long-term studies are expected to detect early phase PC, the king of human cancers.

## Supplementary Materials

Supplementary materials can be found at https://www.mdpi.com/2075-4418/9/4/169/s1.

## Abbreviations

CAPS	International Cancer of the Pancreas Screening Consortium
EUROPAC	European Registry of Hereditary Pancreatitis and Familial Pancreas Cancer
EUS	endoscopic ultrasonography
EUS-FNA	endoscopic ultrasonography-guided fine needle aspiration
FAMMM	familial atypical multiple mole melanoma
FAP	familial adenomatous polyposis
FaPaCa	German National Case Collection for Familial Pancreatic Carcinoma
FDR	first-degree relative
FPC	familial pancreatic cancer
HBOC	hereditary breast ovarian cancer syndrome
HP	hereditary pancreatitis
HR	homologous recombination
HRI	high-risk individual
IPMN	intraductal papillary mucinous neoplasm
JFPCR	Japanese Familial Pancreatic Cancer Registry
LS	Lynch syndrome
MPD	main pancreatic duct
MRCP	magnetic resonance cholangiopancreatography
MRI	magnetic resonance imaging
NFPTR	National Familial Pancreas Tumor Registry
OR	odds ratio
PanIN	pancreatic intraepithelial neoplasm
PARP	poly ADP-ribose polymerase
PC	pancreatic cancer
PJS	Peutz-Jeghers syndrome
RR	relative risk
SIR	standardized incidence ratio
SPC	sporadic pancreatic cancer
FOLFIRINOX	fluorouracil, folic acid, irinotecan, and oxaliplatin
POLO	pancreas cancer olaparib ongoing
